# A Fluorescence‐Based Transient Expression Assay for the Analysis of Upstream Open Reading Frames in Plants

**DOI:** 10.1002/pld3.70163

**Published:** 2026-04-15

**Authors:** Benjamin E. Haas, Faaiza Saif, Emily Bolger, Lynn Doran, Emma Rosales, Abigail Kim, Steven J. Burgess, Stephen P. Long

**Affiliations:** ^1^ Carl R. Woese Institute for Genomic Biology University of Illinois at Urbana‐Champaign Urbana Illinois USA; ^2^ Department of Plant Biology University of Illinois at Urbana‐Champaign Urbana Illinois USA

**Keywords:** agroinfiltration, fluorescence, loop assembly, MoClo, *Nicotiana benthamiana*, transient expression, uORF, upstream open reading frame, VPZ

## Abstract

Upstream open reading frames (uORFs) are regulatory elements present in the 5′ leaders of mRNA that can significantly impact downstream gene expression in eukaryotes. In crop engineering, editing of uORFs can provide an avenue to upregulate expression of native genes without the need to add persistent transgenic copies. Even with genome‐wide methods to identify translated uORFs such as ribosome profiling, their functional characterization depends on validation through reporter gene assays and mutagenesis studies. Current screening methods for plants use luciferases or protoplasts to measure differential gene expression between wild‐type and mutated transcript leaders, which requires tissue processing and/or substrate addition. Here, we present a time‐ and cost‐efficient alternative to investigate transcript leaders by co‐expression of two fluorescent proteins in *Nicotiana benthamiana* leaf tissue and test our assay on genes involved in photoprotection, editing of which could provide a pathway to increase CO_2_ assimilation during sun–shade transitions.

## 1 Introduction

Modifications of gene expression by gene editing play important roles in new plant breeding techniques (Schaart et al. 2016). Recently, upstream open reading frames (uORFs), which are post‐transcriptional regulatory elements residing in the 5′ leader (also known as 5′ untranslated region or 5′ UTR), have emerged as promising candidates for fine‐tuning native gene expression. The introduction or removal of uORFs has been shown to modulate gene products from the primary or main open reading frame (mORF) at the translational level, with minimal impact on transcription, and was used to engineer pathogen resistance, ascorbic acid content, and quantitative traits in crops (Xue et al. 2023; Zhang et al. 2018; Xing et al. 2020; Ai et al. 2022). This method to change native gene expression without the reliance on persistent transgenic copies harbors great potential for the improvement of both food and bioenergy crops (Ort et al. 2015).

Commonly associated with impairing the translation of mORFs, a variety of different regulatory mechanisms for uORFs have been described (Hinnebusch et al. 2016). Typically, scanning ribosomes recognizing uORFs are diverted from mORF activity, and re‐initiation of the ribosome at the mORF is inefficient, depending on the length of the uORF, its location on the 5′ leader, and whether it overlaps with the mORF (von Arnim et al. 2014). Initiation efficiency also depends on the type of start codon and the sequence context, with the canonical AUG start codon in Kozak consensus (5′ A/GNN**AUG**G 3′) being the most efficient for translation initiation. However, noncanonical or near‐cognate start codons that differ from AUG by a single nucleotide can also be recognized by scanning ribosomes, with the likelihood of recognition influenced by the cell's physiological state (Dever et al. 2023).

von Arnim et al. (2014) found that about 30% of genes across 11 different species of angiosperms contain at least one uORF in their 5′ leader, which supports the notion of widespread regulatory potential of uORFs across diverse plant families. The identification of translated uORFs is based on genome‐wide methods, such as ribosomal profiling, complemented by reporter gene assays and mutagenesis studies. Simple and scalable screening methods for 5′ leaders are therefore valuable tools for investigating uORF regulatory functions and translational output.

Several reporter‐based strategies have been developed to study uORF‐mediated regulation in plant cells. Si et al. (2020) utilized a dual‐luciferase approach in plant protoplasts, while Ai et al. (2022) combined GFP and luciferase reporters in *Nicotiana benthamiana* leaf tissue for related applications. More recently, Wu et al. (2022) demonstrated the use of a dual‐fluorescence reporter system in *Arabidopsis* protoplasts to quantify uORF‐mediated translational repression and noise reduction.

The use of protoplasts enables uORF screening across numerous plant species but remains experimentally demanding due to extensive tissue processing and handling. Similarly, while dual‐luciferase assays are highly sensitive and reduce variability between samples by normalizing reporter signals to an internal control, the reaction with luciferin as a substrate requires additional reagent preparation and sample manipulation (Si et al. 2020). Fluorescent proteins (FPs) offer an alternative, but high stability and potential cross‐talk with other pigments make them less attractive for quantitative, transient promoter studies in plants (de Ruijter et al. 2003). However, co‐expression of a second FP can reduce variability during quantification, as in dual‐luciferase assays (Wu et al. 2022). In contrast to protoplast or luciferase‐based assays, we developed a dual‐fluorescence reporter system to screen 5′ leader variants via agroinfiltration of *N. benthamiana* leaf tissue with minimal reagent use and sample handling.

The selected FPs, mNeonGreen and tdTomato, are engineered green and red variants with fast maturation, strong brightness, and high quantum yield (Shaner et al. 2004, 2013). Both have been used independently as well as in combination for localization studies in a variety of cell and tissue types, including *Drosophila* germ cells and human organoids (Agarwal et al. 2023; Beumer et al. 2020; Lin et al. 2022). This indicates their suitability for co‐expression in a plant tissue context, which, to our knowledge, has not yet been tested for this specific fluorophore combination. The dual‐reporter was first validated using 5′ leaders with known regulatory uORFs (Si et al. 2020). Subsequently, we extended the approach to investigate sequences from soybean and cowpea, two crops that hold significant potential for agricultural improvement, with implications for both food security and soil health (Ojiewo et al. 2015). We focused on three candidate genes: *Photosystem II Subunit S* (*PsbS*), *violaxanthin de‐epoxidase* (*VDE*), and *zeaxanthin epoxidase* (*ZEP*), which are core components of the photoprotective mechanism known as nonphotochemical quenching (NPQ) (Müller et al. 2001). Speed of induction and relaxation of NPQ in sun‐to‐shade transitions has been highlighted as a promising target for increasing photosynthetic efficiency and ultimately yield (Long et al. 2022). *PsbS* increased NPQ capacity during field trials when overexpressed transgenically in *Nicotiana tabacum* (Glowacka et al. 2018) and nontransgenically by promoter editing in *Oryza sativa* (Patel‐Tupper et al. 2024). In combination with the *VDE* and *ZEP*, stable overexpression lines exhibited accelerated NPQ relaxation during sun‐shade transitions and ultimately biomass increases in tobacco (Kromdijk et al. 2016) and soybean (De Souza et al. 2022), while similar gains were not achieved in *S. tuberosum* (Lehretz et al. 2022) and *Arabidopsis* (Garcia‐Molina and Leister 2020). To our knowledge, endogenously upregulating all three genes by gene editing has not yet been investigated, but this approach could offer advantages over traditional transgenic methods, such as more precise control over gene expression and reduced regulatory hurdles while providing novel insights into phenotypes associated with faster NPQ relaxation. In this study, we aim to identify repressive uORFs in *PsbS*, *VDE*, and *ZEP* homologs from soybean and cowpea using our dual‐fluorescence assay.

## 2 Materials and Methods

### 2.1 Vector Design and Cloning

Vector design was based on Loop assembly (Pollak et al. 2019) and the MoClo toolkit (Engler et al. 2014), following the common genetic syntax of the Phytobrick standard (Patron et al. 2015). The dual‐fluorescence acceptor RC00214 (Addgene no. 209019) was designed to contain the experimental reporter cassette: CaMV35S promoter (pICH41388), ccdB marker, mNeonGreen, and the CaMV35S terminator (pICH41414). Downstream, the reference reporter cassette consisted of tdTomato controlled by the *octopine synthase* promoter/terminator (pICH88103, pICH41432). We therefore avoided repeating the CaMV35S promoter for both reporter genes to minimize potential transcriptional interference and regulatory cross‐talk between expression cassettes (Yoo et al. 2005). mNeonGreen and tdTomato sequences from FPbase (Lambert 2019) were codon‐optimized for *N. benthamiana* (IDT, USA). *AtBRI1* and *LsGGP2* 5′ leader sequences were adapted from Si et al. (2020). Other 5′ leader sequences were sourced from soybean (Wm82.a6.v1; Espina et al. 2024) and cowpea genomes (v1.2; Liang et al. 2024) on Phytozome (Goodstein et al. 2012): *PsbS* (Glyma.04G249700.1, Vigun09g165900.1), *VDE* (Glyma.19G251000.1, Vigun06g119100.1), and *ZEP* (Glyma.17G174500.1, Vigun03g277500.1). For screening, uORFs were selected based on length (minimum of 9 bp), start codon and Kozak sequence context, with ATG‐uORFs as primary targets followed by non‐ATG start codons in Kozak context. Mutated 5′ leaders had a single start codon replaced with a non‐initiating triplet (e.g., AAA, TTT). The 5′ leaders, mNeonGreen, and tdTomato were commercially synthesized (Twist Bioscience, USA), with internal SapI, BsaI, and BpiI sites removed. The ccdB gene was cloned from pMOD‐B2103 (Addgene no. 91061). The CaMV35S:P19 vector EC27841 was kindly provided by Dr. Paul South.

Vector assembly followed Pollak et al. (2019), and successful 5′ leader insertion was verified via Sanger sequencing (Roy J. Carver Biotechnology Center, IL, USA).

Details on the reaction setup and a full list of 5′ leaders and vectors are provided in Additional File 1.

### 2.2 Plant Materials and Growth Conditions

Wild‐type *N. benthamiana* seeds were directly sown onto soil (Metro‐mix 360, Sun Gro Horticulture, USA) in half‐gallon pots and grown in a plant growth chamber (Conviron, Winnipeg, Canada) under controlled conditions. Chamber conditions were set to 25°C/22°C day/night temperature, 65% relative humidity, 12/12 h day/night cycle, with a daylight intensity of 500 μmol m^2^ s^−1^. Plants were grown 4–6 weeks before infiltration and fertilized with 15‐16‐17 Peat lite (77220, JR Peters INC, USA) every 2 weeks at 0.5 g per liter of water.

### 2.3 Agroinfiltration

C58C1 was transformed by electroporation (Lin 1995) and grown in LB with construct specific antibiotics and Rifampicin (100 μg mL^−1^) at 28°C, shaking at 200 rpm to a target OD_600_ *Agrobacterium tumefaciens* C58C1 was transformed by electroporation (Lin 1995) and grown in LB with construct specific antibiotics and Rifampicin (100 μg ml^−1^) at 28°C, shaking at 200 rpm to a target OD_600_ of 1–1.5.

Infiltration of *N. benthamiana* followed Sparkes et al. (2006), using a modified medium (pH 5.5–5.6) containing 10 MgCl_2_, 10 mM MES, 150 μM acetosyringone (A1104 Phytotech Labs, USA), 0.5% (w/v) glucose, and 0.25% (w/v) MS medium with Gamborg vitamins (M404 Phytotech Labs, USA). The third and fourth youngest, fully expanded leaves were infiltrated at up to four distinct spots without repeating constructs on the same leaf. Plants were kept in darkness under plastic domes overnight post‐infiltration, then exposed to standard growth conditions.

### 2.4 Fluorescence Intensity Measurements

Fluorescence measurements were performed after Pasin et al. (2014). Three days post infiltration, 6 mm diameter leaf discs were excised with a leaf puncher and placed with the abaxial side facing upwards in a black, opaque 96‐well plate (3915, CoStar, USA) supplied with 50 μL sterile water per well. One leaf disc per infiltrated spot was sampled and treated as independent biological replicate. Fluorescence intensity measurements were conducted immediately after sampling with a monochromator‐based, multiwell plate reader (Spectramax M5, Molecular Devices, San Jose, USA). Wavelengths were initially set to reported excitation and emission maxima from literature and adapted according to results from sweep measurements until the background signal was minimal (<5 RFU). Excitation and emission wavelengths (ex/em) were set to 490/520 nm (emission cutoff 515 nm) for mNeonGreen and 540/595 nm (emission cutoff 590 nm) for tdTomato respectively. RFU values were blank corrected against a nonfluorescent control treatment (CaMV35S:P19). Samples with values below a threshold of 1 RFU after blank correction were excluded from the analysis.

### 2.5 Stereomicroscopy and Image Acquisition

Excised leaf discs were placed on a drop of water with the abaxial side facing upwards on an object slide. The samples were imaged using a stereomicroscope (AxioZoom.V16, NA = 0.25, ZEISS, Germany) via widefield fluorescence without a cover slide. Bandpass filters for GFP and RFP imaging were 38 HE (excitation BP 470/40, beamsplitter FT 495, emission BP 525/50; ZEISS, Germany) and 31, respectively (ex. BP 565/30, beamsplitter FT 585, em. BP 620/60; ZEISS, Germany). Images were taken with a monochromatic high‐resolution camera (AxioCam HRm, ZEISS, Germany), 300 ms exposure. Postacquisition processing was done with software supplied by the manufacturer (Zen Blue 2.5, ZEISS, Germany) and included colorization, window, and level adjustments. Levels were kept constant for each channel between acquisitions.

### 2.6 Gene Expression by RT‐qPCR

RT‐qPCR was performed using *N. benthamiana* leaf tissue collected 3 days after agroinfiltration. Biological replicates consisted of total RNA extracted from four independent infiltration spots using the same construct (*n* = 4). For each biological replicate, three technical replicates were prepared by synthesizing cDNA from the same total RNA extract in three separate reactions. First‐strand cDNA synthesis was carried out using SuperScript III (Invitrogen, USA) and oligo (dT) primers. Quantitative PCR was performed with SsoAdvanced Universal SYBR Green Supermix (Bio‐Rad Laboratories, USA) on a CFX96 Real‐Time System (Bio‐Rad Laboratories, USA). Further details on reaction setup are provided in Additional File 1.

### 2.7 Statistical Analysis

Data figures and statistical analyses were made with RStudio 2023.06.0 + 421 “Mountain Hydrangea” Release (2023‐06‐05) using R version 4.2.3 (R Core Team 2023). A detailed list of R packages and their citations is provided in Additional File 2. Schematics and figure formatting were performed with vector graphics software (Adobe Illustrator 27.0.1, Adobe, USA). A mimimum sample size of 8 was chosen for the transient expression assays based on a power calculation using the non‐parametric bootstrap method for two‐sample *t*‐testing (Efron 1979), with a predetermined effect size corresponding to a 50% increase in signal and common error thresholds. The effect size was calculated using the standard deviation from dual‐fluorescence assays with the WT 5′ leaders from *AtBRI1* or *LsGGP2* (Additional File 2). Multiple testing correction followed Benjamini and Hochberg (1995).

## 3 Results

### 3.1 Usability of mNeonGreen and tdTomato for a Dual‐Fluorescence Reporter

**FIGURE 1:**
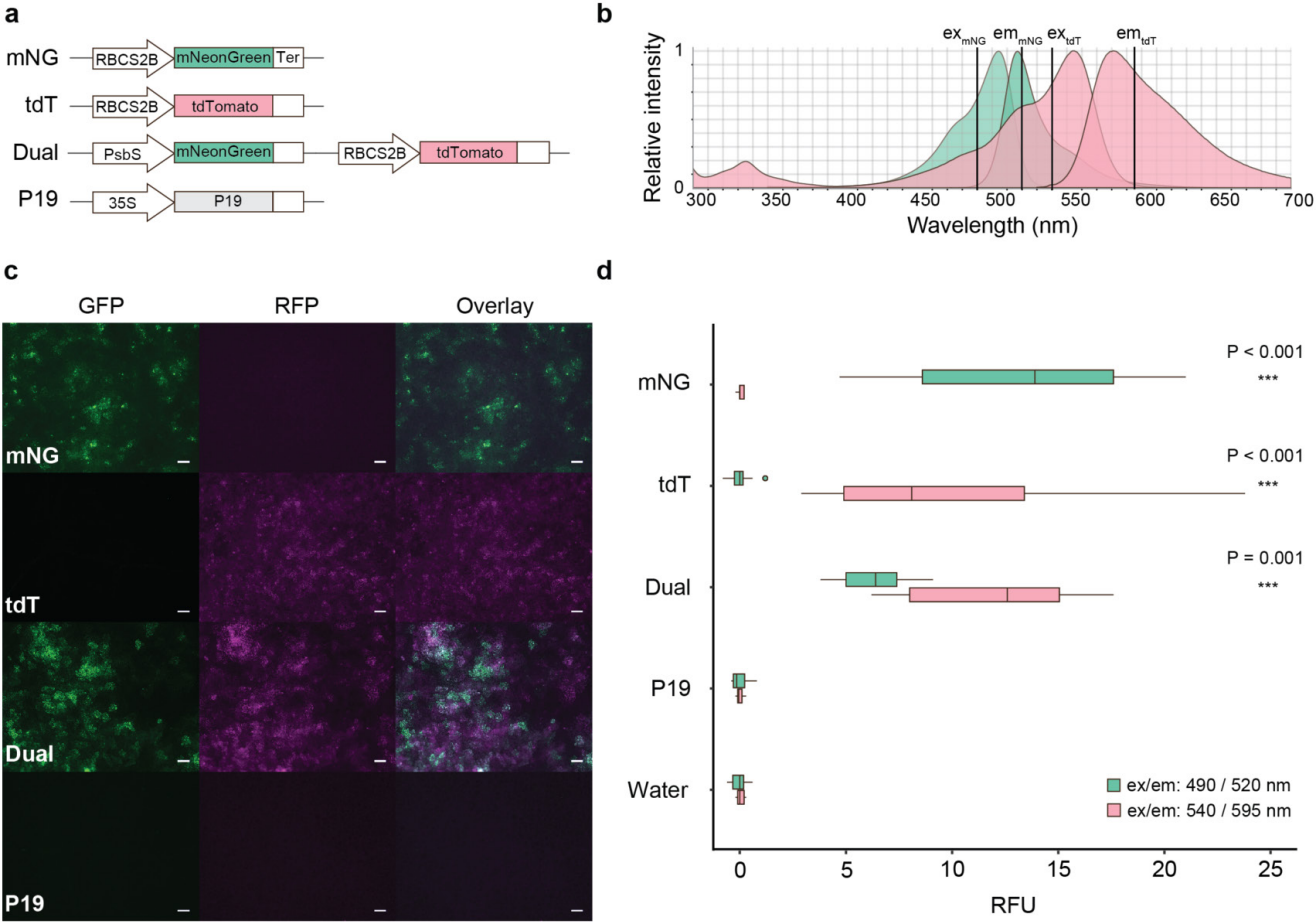
Co‐expression of mNeonGreen and tdTomato in *Nicotiana benthamiana*  Co‐expression of mNeonGreen and tdTomato in *Nicotiana* *benthamiana*. (a) Schematic of fluorescent reporters used during agroinfiltration of *N. benthamiana*: mNeonGreen (mNG), tdTomato (tdT), combined mNeonGreen‐tdTomato (dual), and a negative control (P19). (b) Overlay of published ex/em spectra of mNeonGreen and tdTomato with selected wavelengths used during plate reader measurements. The spectra were formatted from FPbase.org [1] with permission from the authors. (c) Fluorescence stereomicroscopy of excised leaf‐discs with transiently expressed reporters, 3‐day post infiltration, scale bar: 200 μm. (d) Two‐channel fluorescence, 96‐well plate reader measurements of agroinfiltrated leaf discs and control wells containing only water, 3‐day post infiltration. Wilcoxon rank sum test, *n* = 12, ***p < 0.001. RFU, relative fluorescent units.

To test optimal measurement conditions for mNeonGreen and tdTomato, both proteins were expressed transiently in *N. benthamiana* leaf tissue via agroinfiltration, either independently or combined on a single T‐DNA, using the RNA silencing suppressor p19 as a nonfluorescent negative control (Figure 1A). Fluorescence was analyzed by stereo‐microscopy and quantified via plate reader. Adjusted excitation/emission wavelengths during plate reader measurements (Figure 1B) resulted in selective fluorescence detection with minimal background signal between channels (Figure 1D). In this preliminary dual‐reporter configuration, which used the *RbcS2A* promoter for tdTomato and the *PsbS* promoter for mNeonGreen, mNeonGreen fluorescence levels were reduced relative to the corresponding single‐reporter constructs, consistent with differences in promoter usage. In addition, tdTomato signal intensities were also reduced in the dual vector, likely reflecting increased transcriptional load associated with co‐expression. During microscopy, we also observed little background autofluorescence and minimal spectral bleed‐through of mNeonGreen into the RFP channel and of tdTomato into the GFP channel (Figure 1C). These results indicate the suitability of mNeonGreen and tdTomato for co‐expression in *N. benthamiana* and quantitative analysis using a multi‐well plate reader.

**FIGURE 2:**
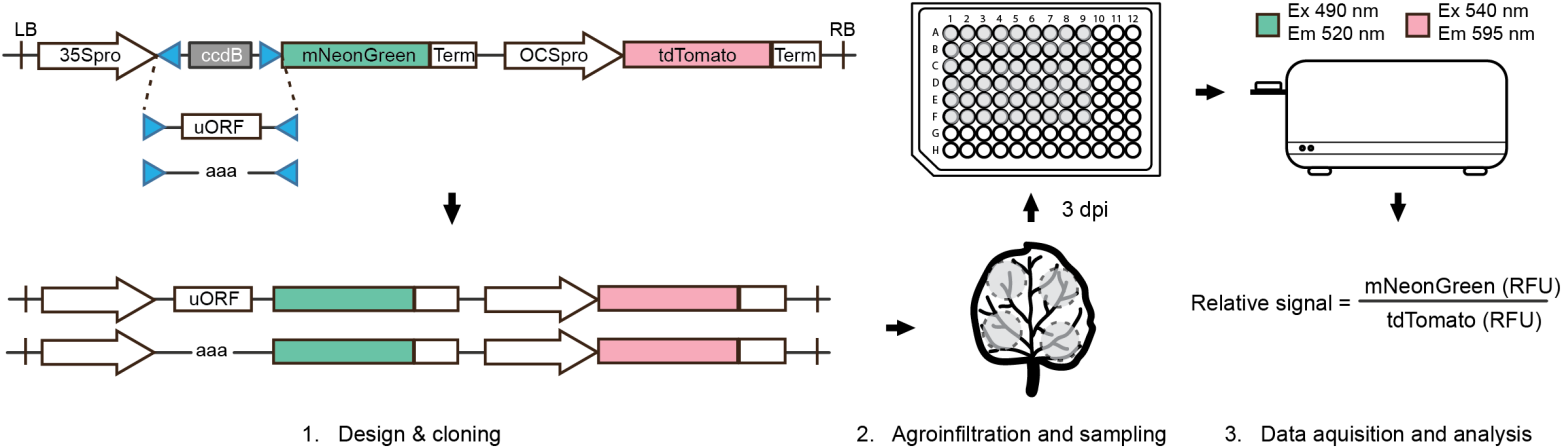
A dual‐fluorescence based assay for the evaluation of transcript leaders. Wild‐type and mutated 5′ leaders are cloned into the acceptor backbone in one step via BpiI, followed by agroinfiltration in *N. benthamiana*, leaf disc excision, and two‐channel fluorescence quantification in a 96‐well plate reader 3‐day postinfiltration. RFU, relative fluorescent units.

A dual‐fluorescence acceptor backbone (Addgene no. 209019) was designed, combining both reporter genes on a single vector and utilizing the CaMV35S promoter to drive expression of the experimental reporter mNeonGreen, while tdTomato served as the reference reporter under control of the *octopine synthase* (OCS) promoter. Insertion of a 5′ leader of interest via BpiI replaces the ccdB bacterial marker located between the CaMV35S promoter and the mNeonGreen coding sequence (Figure 2). The OCS promoter was selected for the reference reporter because it exhibited low variability across infiltrations (Additional Figure 1) and to minimize potential regulatory interference associated with duplicated promoter usage.

### 3.2 Reporter Validation With Known Functional uORFs

**FIGURE 3:**
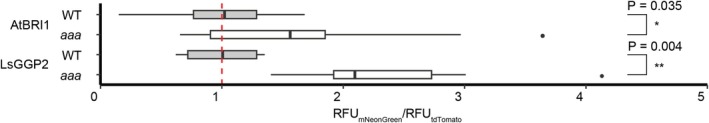
Assay validation with known uORFs after Si et al. (2020). Normalized, relative fluorescence signals of wild‐type and mutated 5′ leaders from *A. thaliana* BRI1 and *L. sativa* GGP2. Average wild‐type levels are marked by the red, dashed line. Nonparametric bootstrap for two‐sample *t*‐testing, *n* = 13–14, *p < 0.05, **p < 0.01. RFU, relative fluorescent units.

In order to validate our method for the screening of regulatory uORFs, we tested 5′ leaders after Si et al. (2020). *Arabidopsis thaliana* *BRI1* and *Lactuca sativa* *GGP2* exhibit strong translational derepression of the mORF when a single uORF is disrupted (Zhang et al. 2018). For *AtBRI1*, this constituted elimination of a 21‐bp non‐overlapping ATG‐uORF and for *LsGGP2*, a conserved peptide ACG‐uORF, also non‐overlapping with the mORF, by replacing the start codon with AAA. The same 5′ leaders were inserted into our reporter backbone, agroinfiltrated and fluorescence measured as shown in Figure 2. Fluorescent signal ratios from 13 to 14 independently infiltrated plants increased on average by almost 70% for mutated *AtBRI1* and over 160% for *LsGGP2* (Figure 3). These results indicate the usability of our reporter for the screening of uORFs in *N. benthamiana* leaf tissue. Power analysis of a nonparametric bootstrap for two‐sample *t*‐testing (Efron 1979), using common error thresholds (α = 0.05, β = 0.2), gave a sample size of *n* = 11 to observe a 50% signal ratio increase for a 5′ leader with a standard deviation equivalent to AtBRI1‐WT (Additional Figure 3). Under the same thresholds, the minimum sample size for a 5′ leader with a spread equivalent to LsGGP2‐WT was *n* = 6 (Additional Figure 4). Based on these results, we opted for a minimum sample size of 8 independently infiltrated leaves per treatment.

### 3.3 Screening uORFs in NPQ‐Related Genes

**FIGURE 4:**
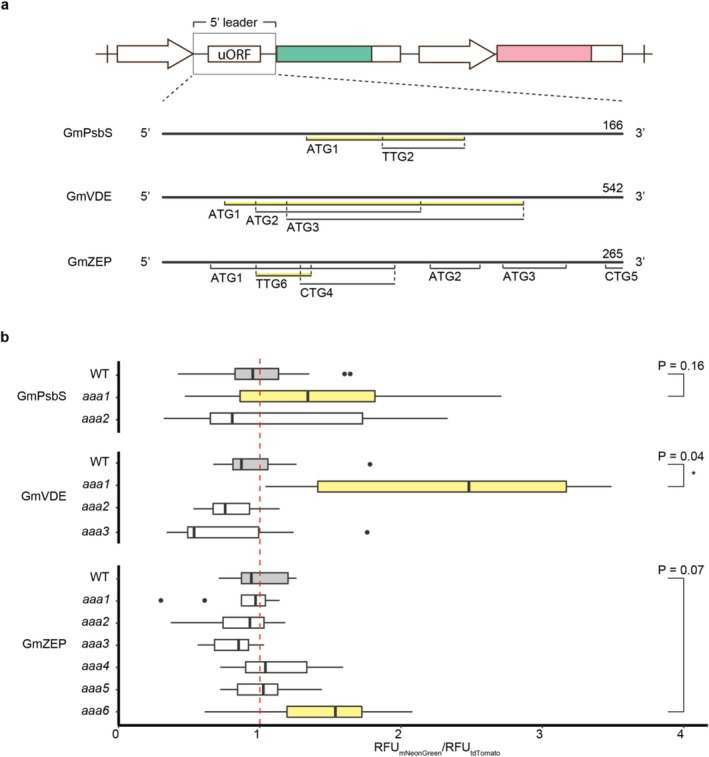
Screening uORFs in transcript leaders from soybean. (a) Schematic of wild‐type, *G. max PsbS*, *VDE* and *ZEP* 5′ leaders inserted into the dual‐fluorescence acceptor with selected uORF candidates. Putative uORFs highlighted in yellow resulted in the largest, relative changes in fluores‐ cence after start codon replacement. 5′ leader length is indicated in base‐pairs. (b) Mean‐normalized, relative fluorescence signals of wild‐type (WT, gray) and mutated 5′ leaders (white/yellow). Data were normalized to the average mNeonGreen/tdTomato RFU ratio of the respective WT control, marked by the red, dashed line. Nonparametric bootstrap for two‐sample *t*‐testing, *n* = 8–12, *p < 0.05. RFU, relative fluorescent units.

**FIGURE 5:**
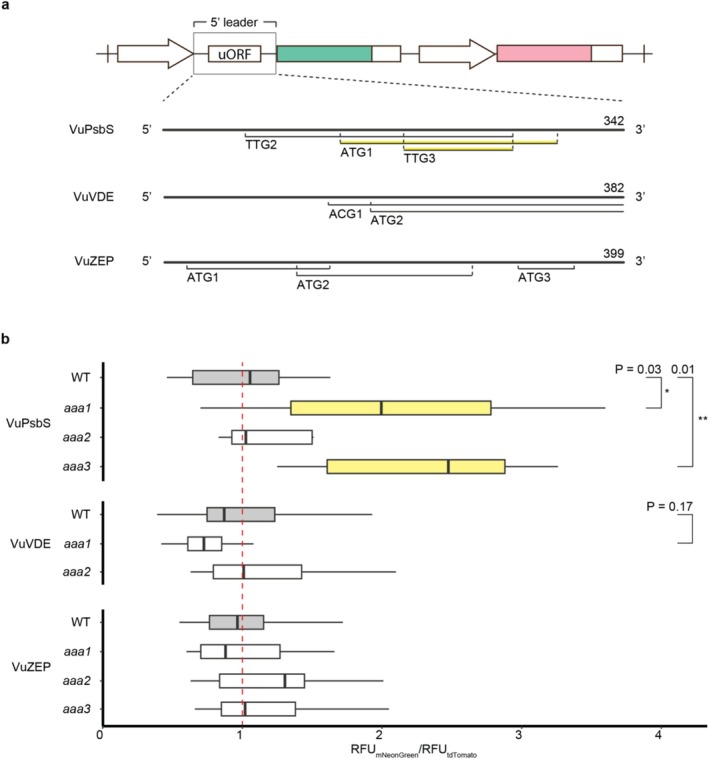
Screening uORFs in transcript leaders from cowpea. (a) Schematic of wild‐type, *V. unguiculata PsbS*, *VDE* and *ZEP* 5′ leaders inserted into the dual‐fluorescence acceptor with selected uORF candidates. Putative uORFs highlighted in yellow resulted in the largest, relative changes in fluorescence after start codon replacement. 5′ leader length is indicated in base‐pairs. (b) Normalized, relative fluorescence signals of wild‐type (WT, gray) and mutated 5′ leaders (white/yellow). Data were normalized to the average mNeonGreen/tdTomato RFU ratio of the respective WT control, marked by the red, dashed line. Nonparametric bootstrap for two‐sample *t*‐testing, *n* = 8–12, *p < 0.05, **p < 0.01. RFU, relative fluorescent units.

**FIGURE 6:**
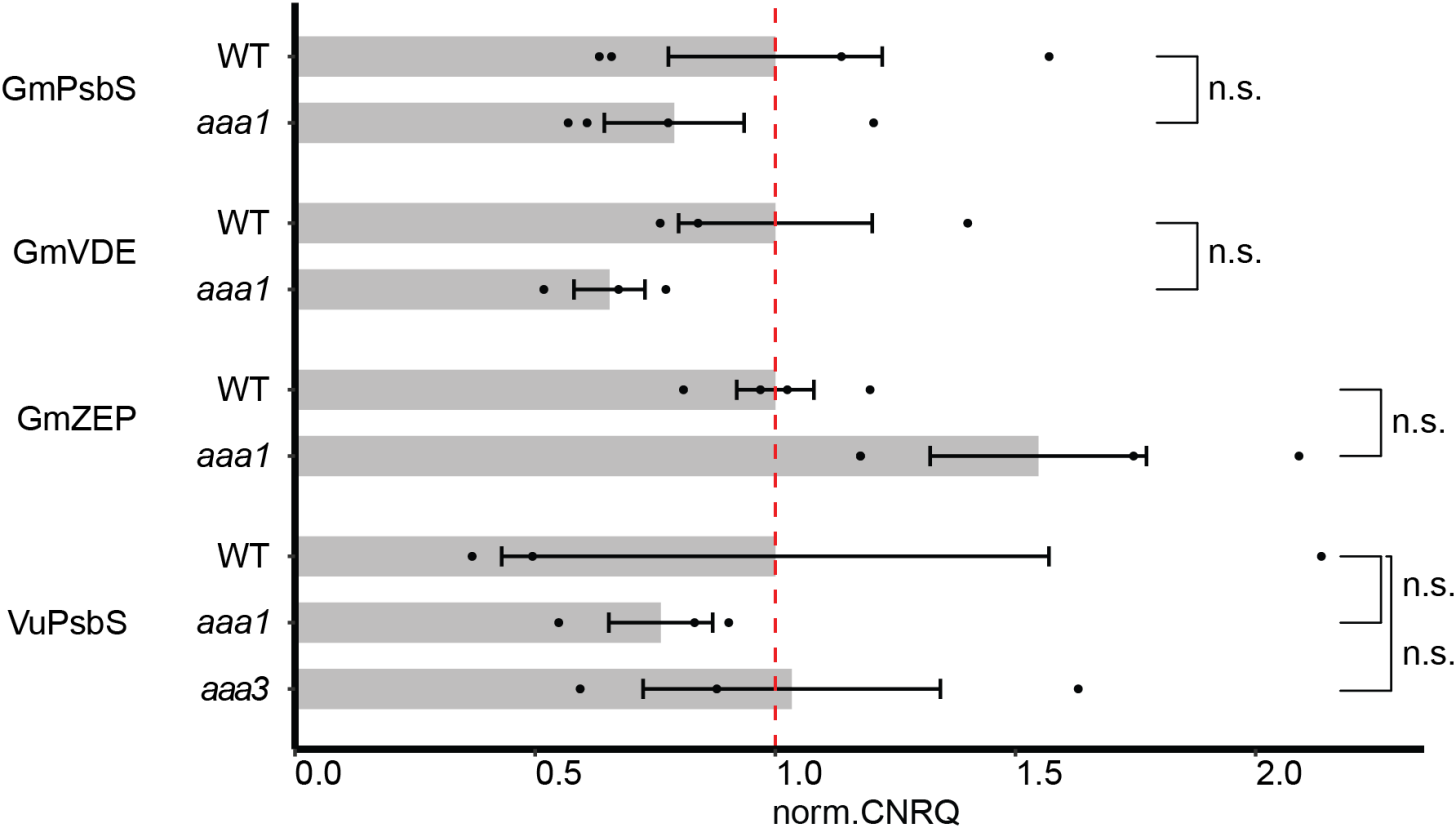
Transcript level comparison. Normalized mRNA levels of WT and uORF disrupted 5′ leaders with largest changes in signal during the dual‐fluorescence assay. Bar height represents the normalized mean CNRQ value and error bars signify the standard error of the mean (s.e.m). For each gene, the mean mRNA level was normalized to those of the corresponding WT 5′ leader (red dashed line). Kruskal–Wallis, Dunn's *post hoc* test, *n* = 3*–*4; n.s., no significant difference (*p <* 0.5).

Although *Glycine max* and *Vigna unguiculata* have multiple homologs for *PsbS*, *VDE*, and *ZEP* (with only one found for *VuVDE*), our screen focused on a single homolog for each gene. Multiple uORF candidates in the annotated 5′ leaders were identified based on cognate or near‐cognate start codons and a reading frame length of over 9 bp. The transcript leader of the selected *GmPsbS* contained one ATG (GmPsbS‐ATG1) and one non‐ATG reading frame (GmPsbS‐TTG2) in a Kozak consensus sequence context with a purine at −3 and a G at +4 . When tested in our reporter assay, although the mutation of ATG1 (GmPsbS‐*aaa1*) exhibited a 43% higher signal ratio compared to WT, the *p*‐value was outside the 5% threshold. The output of GmPsbS‐*aaa2* remained similar to WT (Figure 4). For the selected *GmVDE*, the intronic transcript leader included three ATG uORF candidates with their start codons in close proximity of one another. The first of which (GmVDE‐*aaa1*) gave a signal ratio increase of 136% after disruption. In the *GmZEP* transcript leader, mutation of multiple canonical ATG start codons did not yield greater change in reporter output compared to the WT. However, elimination of a small, 21 bp, TTG‐uORF candidate (GmZEP‐*aaa6*) increased the reporter output by almost 40%, albeit at a p‐value of 0.07 (Figure 4). In cowpea *PsbS*, the three closest and longest uORFs to the mORF were assayed, one with an ATG and two with TTG start codons in Kozak sequence context (VuPsbS‐ATG1, VuPsbS‐TTG2 and VuPsbS‐TTG3 respectively). VuPsbS‐*aaa1* and VuPsbS‐*aaa3* resulted in signal increases of 108% and 130% compared to WT respectively. The transcript leader of *VuVDE* contained two long, overlapping uORFs (VuVDE‐ACG1 and VuVDE‐ATG2) which were selected for mutation. The elimination of the ACG uORF in Kozak context (VuVDE‐*aaa1*) resulted in a modest decrease of 26% in relative fluorescence, though not statistically significant, whereas VuVDE‐*aaa2* did not deviate considerably from the WT. As for the *VuZEP* 5′ leader, none of the three ATG‐uORF candidates that were disrupted resulted in strong changes in signal (Figure 5). Treatments with the largest differences to WT were selected for a comparison of relative transcript levels through RT‐qPCR. Transcript levels from most mutated 5′ leaders were slightly reduced as compared to WT, only GmZEP‐*aaa6* exhibited higher levels, though none of the differences were statistically significant (Figure 6).

## 4 Discussion

Here, we present an easy‐to‐use transient expression assay for the functional screening of uORFs in *N. benthamiana* and provide a dual‐fluorescence acceptor backbone for 5′ leader insertion in one step. This streamlined assembly relies solely on BpiI, and therefore, only a single restriction site must be absent from the 5′ leader, which reduces sequence constraints compared to traditional cloning methods. Furthermore, because the suggested 5′ and 3′ extensions follow the Phytobrick standard (Patron et al. 2015), 5′ leaders can be readily added to your MoClo library (Engler et al. 2014). The entire workflow, from reporter cloning to agroinfiltration and fluorescence measurement, can be accomplished within 2 weeks.

We demonstrated that co‐expression of the two FPs mNeonGreen and tdTomato in *N. benthamiana* enabled selective quantification from excised leaf‐discs in a monochromator‐based plate reader. Compared to luciferase‐based uORF assays, our approach utilizes fewer consumables (Ai et al. 2022) and avoids the need for protoplast isolation (Si et al. 2020), though it generally requires a larger sample size due to higher variability. However, acquiring a bigger sample size requires little additional effort as agroinfiltration of *N. benthamiana* is scalable. When comparing the results from *AtBRI1* and *LsGGP2*, the 0.7‐ and 1.6‐fold increases in relative fluorescence of mutated transcript leaders were less pronounced compared to the 1.5‐ and 6‐fold increases in LUC/REN activity observed by Si et al. (2020), respectively. This discrepancy may be attributed to higher sensitivity and lower background noise of chemiluminescence compared to fluorescence.

In this study, candidate uORFs were selected manually based on established principles of translation initiation, including the presence of cognate or near‐cognate start codons and favorable Kozak context. Recently, predictive tools such as TISCalling (Yen et al. 2025) have been developed to identify translated initiation sites in 5′ leaders by integrating ribosome profiling and transcriptomic data within a machine learning–based framework. While not applied here, such tools could be readily incorporated to inform uORF selection and prioritize candidates for functional screening.

Using our assay, we identified three uORF candidates (GmVDE‐ATG1, VuPsbS‐ATG1, and VuPsbS‐TTG3) with inhibitory effects on the mORF. Two additional putative uORFs (GmPsbS‐ATG1 and GmZEP‐TTG6) indicated a repressive character, albeit with much lower confidence. Though not statistically significant, mRNA levels of GmZEP‐*aaa1* were elevated so transcriptional effects should not be ruled out. Additional experiments are required to confirm the functional character of the uORFs outside the context of transient expression, for example, by targeted knock‐out in stable lines. When Patel‐Tupper et al. (2024) achieved nontransgenic overexpression of *PsbS* in rice, the authors highlighted that much of the observed phenotypic variation across mutant lines was associated with indels in the transcript leader. Our findings could offer an explanation for these results and may warrant a closer look at conserved uORF regulation of *PsbS* across plant species. We also emphasize that Xue et al. (2023) demonstrated how phenotypic changes resulting from uORF insertion or elongation closely aligned with outcomes from prior reporter assays. Building on these studies, we hope that the presented workflow expands the molecular toolbox for screening 5′ leaders *in planta*.

## 5 Conclusion

This study presents a time‐efficient transient expression assay for the functional screening of uORFs in *N. benthamiana*. The assay offers a rapid cloning workflow, utilizing a dual‐fluorescence reporter to quantify the expression strength of transcript leaders of interest. Our identification of several candidate uORFs with inhibitory effects underscores the utility of this approach for characterizing potential regulatory elements. These findings contribute to the expanding body of research on uORF‐mediated gene regulation and provide a foundation for further investigations into gene editing of *PsbS*, *VDE*, and *ZEP*.

## Author Contributions

BEH and SPL conceived and designed the experiments. BEH, FS, EB, LD, ER, and AK performed the experiments. BEH, FS, and LD analyzed the data. BEH, FS, LD, SJB, and SPL wrote the manuscript. All authors read and approved the final manuscript.

## Funding

This work was supported by the Bill and Melinda Gates Foundation, the Foundation for Food and Agriculture Research, and the UK Government’s Department for International Development under award number OPP1172157, the Bill and Melinda Gates Agricultural Innovations under investment ID 57248, and the Center for Advanced Bioenergy and Bioproducts Innovation (CABBI) at the University of Illinois, funded by the US Department of Energy, Office of Science, Biological and Environmental Research Program under Award Number DE‐SC0018420.

## Conflicts of Interest

The authors declare no conflicts of interest.

## Supporting information




**Additional File 1:** Sequence information and additional methods.

**Additional File 2:** Testing of tdTomato reference cassettes and power analysis.

## Data Availability

All data supporting the results of this study are included within the article and the accompanying Supporting Information. The dual‐fluorescence backbone is available through Addgene no. 209019. R scripts used for analysis are openly available on Figshare at https://doi.org/10.6084/m9.figshare.31828477. Other materials used in this study are available from the corresponding author upon request.
