# Supplementary material for: A Fluorescence‐Based Transient Expression Assay for the Analysis of Upstream Open Reading Frames in Plants
Source: Plant Direct. 2026 Apr 15;10(4):e70163. doi: 10.1002/pld3.70163 (PMC13084149; doi:10.1002/pld3.70163)
Supplement: Supplementary file 1 — Additional File 1: Sequence information and additional methods. [file PLD3-10-e70163-s002.docx]

Additional File 1

**Additional Note 1: 5' leader sequences**

Sequences of *de novo* synthesized wild-type and mutated transcript leaders. Assemble into the dual reporter RC00214 (Addgene #209019) for transient expression assays or MoClo level 0 (5U/5U+NT1) acceptor pICH41246 (Addgene #47992). 5’ and 3’ extensions are shown in blue, including optional DraIII and required BpiI restriction sites (lower case). The sequence retained in the acceptor plasmid after assembly is indicated in upper case, selected uORFs start codons are marked in bold, mutated start codons in orange.

L-1M-U-AtBRI1

cactctgtgaagacttTACTGTCTTTTGACTCTCTTCTCTCTCTCCTCTCTTTCTGCTTTCCTCAATCTCTCTCTTTCTATCTCTAGAGCTTCCACTTCCTCTCTA**ATG**GTGGAACCAAAACCCTAGATTCCCCCTTTCATCTTCTCTACTTCCCACACTTTTCTCTCTCACAAACTCTTGAGAAAATGaagtcttccacgaagtg

L-1M-U-AtBRI1-AAA

cactctgtgaagacttTACTGTCTTTTGACTCTCTTCTCTCTCTCCTCTCTTTCTGCTTTCCTCAATCTCTCTCTTTCTATCTCTAGAGCTTCCACTTCCTCTCTAAAAGTGGAACCAAAACCCTAGATTCCCCCTTTCATCTTCTCTACTTCCCACACTTTTCTCTCTCACAAACTCTTGAGAAAATGaagtcttccacgaagtg

L-1M-U-LsGGP2

cactctgtgaagacttTACTAACCACTTTTCCACCATTCATTCGCATCATCAATCTCATTCAAAGTTTCAAACAAATCACCCTCTTCCCCTTTGCTGATTGACCTACTGCTGCCGGATAATCGTCATCGAATTAGGAGAAACAGAGTCTACCCTTGGGGTTGCCTGCTAGAGAGGAACGGGTTGGGGTAAAACCCTGGAACGACAAGTTGCAGACATC**ACG**GCTATACACGGAGCAACGCGGCCACTCGTTCATGTCCGATCAGTACGGCGGAAGGGTTCAATAACTATTGAGAGCAACCCTTCGACGCACGGCGGACGTGGCGCTTTGCCTTCCGAAGGCGGTAGCCCATCCGACCTTCTCTTCCTCGCCGGCGGTGGTTCATCCTCCTTCCTATTCCTCGTAGCTTAGGGTATATTAGGTCTAGACTTATACGCATATATCCCATACCTGTTATTATCGTTTAATTTTCAAGATCTGCTTTCTTCATCGCTCGTATCCGTTTAATCAACCAACCAATCGAAGAACAATGaagtcttccacgaagtg

L-1M-U-LsGGP2-AAA

cactctgtgaagacttTACTAACCACTTTTCCACCATTCATTCGCATCATCAATCTCATTCAAAGTTTCAAACAAATCACCCTCTTCCCCTTTGCTGATTGACCTACTGCTGCCGGATAATCGTCATCGAATTAGGAGAAACAGAGTCTACCCTTGGGGTTGCCTGCTAGAGAGGAACGGGTTGGGGTAAAACCCTGGAACGACAAGTTGCAGACATCAAAGCTATACACGGAGCAACGCGGCCACTCGTTCATGTCCGATCAGTACGGCGGAAGGGTTCAATAACTATTGAGAGCAACCCTTCGACGCACGGCGGACGTGGCGCTTTGCCTTCCGAAGGCGGTAGCCCATCCGACCTTCTCTTCCTCGCCGGCGGTGGTTCATCCTCCTTCCTATTCCTCGTAGCTTAGGGTATATTAGGTCTAGACTTATACGCATATATCCCATACCTGTTATTATCGTTTAATTTTCAAGATCTGCTTTCTTCATCGCTCGTATCCGTTTAATCAACCAACCAATCGAAGAACAATGaagtcttccacgaagtg

L-1M-U-GmPSBS (Glyma.04G249700.1)

cactctgtgaagacTTTACTAACGTGGTCACTCTAGTTGCCTGTCATCAACTATTACACCACATCCACACCCTTGTTAACTTAAAG**ATG**TTATTCCCATTCTATCACATT**TTG**GTAATCTCTTAACATATAGAACACTACCCTGAACATAGTAAAGAAGCCAGTGCTAGGATTTGTTAGTGAAGCAAATGAAgtcttccacgaagtg

L-1M-U-GmPSBS-AAA1

cactctgtgaagacTTTACTAACGTGGTCACTCTAGTTGCCTGTCATCAACTATTACACCACATCCACACCCTTGTTAACTTAAAGAAATTATTCCCATTCTATCACATTTTGGTAATCTCTTAACATATAGAACACTACCCTGAACATAGTAAAGAAGCCAGTGCTAGGATTTGTTAGTGAAGCAAATGAAgtcttccacgaagtg

L-1M-U-GmPSBS1-AAA2

cactctgtgaagacTTTACTAACGTGGTCACTCTAGTTGCCTGTCATCAACTATTACACCACATCCACACCCTTGTTAACTTAAAGATGTTATTCCCATTCTATCACATTAAAGTAATCTCTTAACATATAGAACACTACCCTGAACATAGTAAAGAAGCCAGTGCTAGGATTTGTTAGTGAAGCAAATGAAgtcttccacgaagtg

L-1M-GmZEP3u (Glyma.17G174500.1)

cactctgtgaagacTTTACTGAGTTCACCCCTCAAACGTTAATTAATTACAAGGCTT**ATG**TCTATTTTCAGTATTTTTGACATCTTCGTTCTCCTGATTTTTGTTGGCTTTAACTTTAAACCACCAATTCTGTTCCCCTTGTTTCTTCATCATTAGATTTTAAGTGCC**ATG**CATTGATATAATATAAATATATAACTTCATTTAATTA**ATG**CAAGCTATTGAGTTTTGAGTCGAATCTATTAGCCACAAAACACACACACACATATTCTCACACAAACTGCAACCAATGAAgtcttccacgaagtg

L-1M-GmZEP3u-AAA1

cactctgtgaagacTTTACTGAGTTCACCCCTCAAACGTTAATTAATTACAAGGCTTAAATCTATTTTCAGTATTTTTGACATCTTCGTTCTCCTGATTTTTGTTGGCTTTAACTTTAAACCACCAATTCTGTTCCCCTTGTTTCTTCATCATTAGATTTTAAGTGCCATGCATTGATATAATATAAATATATAACTTCATTTAATTAATGCAAGCTATTGAGTTTTGAGTCGAATCTATTAGCCACAAAACACACACACACATATTCTCACACAAACTGCAACCAATGAAgtcttccacgaagtg

L-1M-GmZEP3u-AAA2

cactctgtgaagacTTTACTGAGTTCACCCCTCAAACGTTAATTAATTACAAGGCTTATGTCTATTTTCAGTATTTTTGACATCTTCGTTCTCCTGATTTTTGTTGGCTTTAACTTTAAACCACCAATTCTGTTCCCCTTGTTTCTTCATCATTAGATTTTAAGTGCCAAACATTGATATAATATAAATATATAACTTCATTTAATTAATGCAAGCTATTGAGTTTTGAGTCGAATCTATTAGCCACAAAACACACACACACATATTCTCACACAAACTGCAACCAATGAAgtcttccacgaagtg

L-1M-GmZEP3u-AAA3

cactctgtgaagacTTTACTGAGTTCACCCCTCAAACGTTAATTAATTACAAGGCTTATGTCTATTTTCAGTATTTTTGACATCTTCGTTCTCCTGATTTTTGTTGGCTTTAACTTTAAACCACCAATTCTGTTCCCCTTGTTTCTTCATCATTAGATTTTAAGTGCCATGCATTGATATAATATAAATATATAACTTCATTTAATTAAAACAAGCTATTGAGTTTTGAGTCGAATCTATTAGCCACAAAACACACACACACATATTCTCACACAAACTGCAACCAATGAAgtcttccacgaagtg

L-1M-GmVDE1u (Glyma.19G251000.1)

cactctgtgaagacTTTACTCCTTATTGGACTTTAGAATATTGAGTCCATTTGCAAGGAAATTGGAAGCAAATAGAGGCGTCGACGTGTCTGTTGAGGTTCCAGTGAGTGACGGGTCTGTTTGCAGTCCACACAAACACCATCATCATC**ATG**TTCTTATCTC**ATG**TT**ATG**CGACGGCGACTCTTCTTTCTTCTTCTTCCTCTTCTTCCTCACATTTTCTTATTCTATTTCTCATTTTACCTGCACTTGGCCTCTTCTACGCCGCAAGGCATGTTTCCAACTTTGCTCATTCTAAATTATATCTCTGCCCTTAACTACTATTATAAACTAAATTTTGTAACTTGGCTAATGAAAACAAGTGGGTATGCAATATTCTTGGACAATTATATTTTCCTTTTATTATAGAGTCTTTAGTATTCCATTTTGGGCTATGATTACCTCTGCATTCTAAATGATATCCTGTTGATCTTGTTGATTTGGTTTTCACAGTATCAGGTTACATTTGGAGTTAAGACATATACATATATTAATATATTATTATTATTATTATATTATATGTATATAATGAAgtcttccacgaagtg

L-1M-GmVDE1u-AAA1

cactctgtgaagacTTTACTCCTTATTGGACTTTAGAATATTGAGTCCATTTGCAAGGAAATTGGAAGCAAATAGAGGCGTCGACGTGTCTGTTGAGGTTCCAGTGAGTGACGGGTCTGTTTGCAGTCCACACAAACACCATCATCATCAAATTCTTATCTCATGTTATGCGACGGCGACTCTTCTTTCTTCTTCTTCCTCTTCTTCCTCACATTTTCTTATTCTATTTCTCATTTTACCTGCACTTGGCCTCTTCTACGCCGCAAGGCATGTTTCCAACTTTGCTCATTCTAAATTATATCTCTGCCCTTAACTACTATTATAAACTAAATTTTGTAACTTGGCTAATGAAAACAAGTGGGTATGCAATATTCTTGGACAATTATATTTTCCTTTTATTATAGAGTCTTTAGTATTCCATTTTGGGCTATGATTACCTCTGCATTCTAAATGATATCCTGTTGATCTTGTTGATTTGGTTTTCACAGTATCAGGTTACATTTGGAGTTAAGACATATACATATATTAATATATTATTATTATTATTATATTATATGTATATAATGAAgtcttccacgaagtg

L-1M-GmVDE1u-AAA2

cactctgtgaagacTTTACTCCTTATTGGACTTTAGAATATTGAGTCCATTTGCAAGGAAATTGGAAGCAAATAGAGGCGTCGACGTGTCTGTTGAGGTTCCAGTGAGTGACGGGTCTGTTTGCAGTCCACACAAACACCATCATCATCATGTTCTTATCTCAAATTATGCGACGGCGACTCTTCTTTCTTCTTCTTCCTCTTCTTCCTCACATTTTCTTATTCTATTTCTCATTTTACCTGCACTTGGCCTCTTCTACGCCGCAAGGCATGTTTCCAACTTTGCTCATTCTAAATTATATCTCTGCCCTTAACTACTATTATAAACTAAATTTTGTAACTTGGCTAATGAAAACAAGTGGGTATGCAATATTCTTGGACAATTATATTTTCCTTTTATTATAGAGTCTTTAGTATTCCATTTTGGGCTATGATTACCTCTGCATTCTAAATGATATCCTGTTGATCTTGTTGATTTGGTTTTCACAGTATCAGGTTACATTTGGAGTTAAGACATATACATATATTAATATATTATTATTATTATTATATTATATGTATATAATGAAgtcttccacgaagtg

L-1M-GmVDE1u-AAA3

cactctgtgaagacTTTACTCCTTATTGGACTTTAGAATATTGAGTCCATTTGCAAGGAAATTGGAAGCAAATAGAGGCGTCGACGTGTCTGTTGAGGTTCCAGTGAGTGACGGGTCTGTTTGCAGTCCACACAAACACCATCATCATCATGTTCTTATCTCATGTTAAACGACGGCGACTCTTCTTTCTTCTTCTTCCTCTTCTTCCTCACATTTTCTTATTCTATTTCTCATTTTACCTGCACTTGGCCTCTTCTACGCCGCAAGGCATGTTTCCAACTTTGCTCATTCTAAATTATATCTCTGCCCTTAACTACTATTATAAACTAAATTTTGTAACTTGGCTAATGAAAACAAGTGGGTATGCAATATTCTTGGACAATTATATTTTCCTTTTATTATAGAGTCTTTAGTATTCCATTTTGGGCTATGATTACCTCTGCATTCTAAATGATATCCTGTTGATCTTGTTGATTTGGTTTTCACAGTATCAGGTTACATTTGGAGTTAAGACATATACATATATTAATATATTATTATTATTATTATATTATATGTATATAATGAAgtcttccacgaagtg

L-1M-U-VuPSBS1u (Vigun09g165900.1)

cactctgtgaagacTTTACTAATAAAATTGAACCAAGAAAATAAAATACAGAGAGCGAAATGAACTAAAGTGAGTGAGTGTACTAAGTTTGAGGCAGGAAACGAAAGGAGATGAATATTATTTTTTGCAAAACCTGAAATGAAAACATTATCACCATACCACAAATAGTG**TTG**GAGACGTGGTCACTCTTGTTGCCTGTCATCAACTATTACACCACATCCACACCCTTGTTAAATTATAG**ATG**GTGTTCCTCTTCTATCATCATT**TTG**GTAAACCCTACCTTAACATATTCAACACTACCATTCCCAACATTCTGAACCAAACCACCGAGGTAAGGTAACACTTCGTTTCATACTCCACCAAATGAAgtcttccacgaagtg

L-1M-U-VuPSBS1u-AAA1

cactctgtgaagacTTTACTAATAAAATTGAACCAAGAAAATAAAATACAGAGAGCGAAATGAACTAAAGTGAGTGAGTGTACTAAGTTTGAGGCAGGAAACGAAAGGAGATGAATATTATTTTTTGCAAAACCTGAAATGAAAACATTATCACCATACCACAAATAGTGTTGGAGACGTGGTCACTCTTGTTGCCTGTCATCAACTATTACACCACATCCACACCCTTGTTAAATTATAGAAAGTGTTCCTCTTCTATCATCATTTTGGTAAACCCTACCTTAACATATTCAACACTACCATTCCCAACATTCTGAACCAAACCACCGAGGTAAGGTAACACTTCGTTTCATACTCCACCAAATGAAgtcttccacgaagtg

L-1M-U-VuPSBS1u-AAA2

cactctgtgaagacTTTACTAATAAAATTGAACCAAGAAAATAAAATACAGAGAGCGAAATGAACTAAAGTGAGTGAGTGTACTAAGTTTGAGGCAGGAAACGAAAGGAGATGAATATTATTTTTTGCAAAACCTGAAATGAAAACATTATCACCATACCACAAATAGTGTTTGAGACGTGGTCACTCTTGTTGCCTGTCATCAACTATTACACCACATCCACACCCTTGTTAAATTATAGATGGTGTTCCTCTTCTATCATCATTTTGGTAAACCCTACCTTAACATATTCAACACTACCATTCCCAACATTCTGAACCAAACCACCGAGGTAAGGTAACACTTCGTTTCATACTCCACCAAATGAAgtcttccacgaagtg

L-1M-U-VuPSBS1u-AAA3

cactctgtgaagacTTTACTAATAAAATTGAACCAAGAAAATAAAATACAGAGAGCGAAATGAACTAAAGTGAGTGAGTGTACTAAGTTTGAGGCAGGAAACGAAAGGAGATGAATATTATTTTTTGCAAAACCTGAAATGAAAACATTATCACCATACCACAAATAGTGTTGGAGACGTGGTCACTCTTGTTGCCTGTCATCAACTATTACACCACATCCACACCCTTGTTAAATTATAGATGGTGTTCCTCTTCTATCATCATT**TTT**GTAAACCCTACCTTAACATATTCAACACTACCATTCCCAACATTCTGAACCAAACCACCGAGGTAAGGTAACACTTCGTTTCATACTCCACCAAATGAAgtcttccacgaagtg

L-1M-U-VuVDE1u (Vigun06g119100.1)

cactctgtgaagacTTTACTAAAAAATAATAAAGAAGATTTTAACTATAGTTTTGTGATAAGATTATGAAGTGGGCCTTGAAAGAAGGTATTGGACTTGTAACATTGAGTCCATTTGCAAGAAAATTGGAAGCAAATTGAAGCGTGGACGTGTGCGGTGTGGTTGCAGAGTGCAGTG**ACG**GGT**ATG**TTTGCAGTCCACACGAACACCATCGTTTTCTTATCCATCTCATCTCATCTCATCCATCCCCGGAACTCTTCTTATGCTACGCCGCCATTTCTCTCTTCTAAATCCTCTTATTCTATTATCTCTTTCACTGTACCTGCAATTCGCCACATATACACCGCAAGATTGAATCTGGAGGTGAGGACATACACATACATATACTGTTGTATTGTATGTATGAATGAAgtcttccacgaagtg

L-1M-U-VuVDE1u-AAA1

cactctgtgaagacTTTACTAAAAAATAATAAAGAAGATTTTAACTATAGTTTTGTGATAAGATTATGAAGTGGGCCTTGAAAGAAGGTATTGGACTTGTAACATTGAGTCCATTTGCAAGAAAATTGGAAGCAAATTGAAGCGTGGACGTGTGCGGTGTGGTTGCAGAGTGCAGTGAAAGGTATGTTTGCAGTCCACACGAACACCATCGTTTTCTTATCCATCTCATCTCATCTCATCCATCCCCGGAACTCTTCTTATGCTACGCCGCCATTTCTCTCTTCTAAATCCTCTTATTCTATTATCTCTTTCACTGTACCTGCAATTCGCCACATATACACCGCAAGATTGAATCTGGAGGTGAGGACATACACATACATATACTGTTGTATTGTATGTATGAATGAAgtcttccacgaagtg

L-1M-U-VuVDE1u-AAA2

cactctgtgaagacTTTACTAAAAAATAATAAAGAAGATTTTAACTATAGTTTTGTGATAAGATTATGAAGTGGGCCTTGAAAGAAGGTATTGGACTTGTAACATTGAGTCCATTTGCAAGAAAATTGGAAGCAAATTGAAGCGTGGACGTGTGCGGTGTGGTTGCAGAGTGCAGTGACGGGTAAATTTGCAGTCCACACGAACACCATCGTTTTCTTATCCATCTCATCTCATCTCATCCATCCCCGGAACTCTTCTTATGCTACGCCGCCATTTCTCTCTTCTAAATCCTCTTATTCTATTATCTCTTTCACTGTACCTGCAATTCGCCACATATACACCGCAAGATTGAATCTGGAGGTGAGGACATACACATACATATACTGTTGTATTGTATGTATGAATGAAgtcttccacgaagtg

L-1M-U-VuZEP1u (Vigun03g277500.1)

cactctgtgaagacTTTACTACTCT**ATG**GGTTTGAATCCTGCTTCAGATTTTCCTACGTGGGCGAGCTTACAACATACACACCACTCCCATCC**ATG**ACCACAATTAAGGTACCAAAATTTGTCTGTCACTGTCACCCAACACTAGCAACCACTGATCTCATTTCTCACACGTTAATTACAAATTACGAACCTTACGTCTAGTTTCATCATCCTCATTCTCCTGATTTTTCTAACCATTAACTTTAAACCACTGATTCTGTTGTCCTTGTTTATTCGTCATTAGATTTTGTTATTTCGATTCATTGTCAAAACTTCAACCTGTAATCGTATAAATATAACTTCACCTGATTA**ATG**CAACCTAGTTTTTGAGTTTTGAGTTGAGCCACACAAGGCACACATTCACACACGCACCAACAACCAATGAAgtcttccacgaagtg

L-1M-U-VuZEP1u-AAA1

cactctgtgaagacTTTACTACTCTAAAGGTTTGAATCCTGCTTCAGATTTTCCTACGTGGGCGAGCTTACAACATACACACCACTCCCATCCATGACCACAATTAAGGTACCAAAATTTGTCTGTCACTGTCACCCAACACTAGCAACCACTGATCTCATTTCTCACACGTTAATTACAAATTACGAACCTTACGTCTAGTTTCATCATCCTCATTCTCCTGATTTTTCTAACCATTAACTTTAAACCACTGATTCTGTTGTCCTTGTTTATTCGTCATTAGATTTTGTTATTTCGATTCATTGTCAAAACTTCAACCTGTAATCGTATAAATATAACTTCACCTGATTAATGCAACCTAGTTTTTGAGTTTTGAGTTGAGCCACACAAGGCACACATTCACACACGCACCAACAACCAATGAAgtcttccacgaagtg

L-1M-U-VuZEP1u-AAA2

cactctgtgaagacTTTACTACTCTATGGGTTTGAATCCTGCTTCAGATTTTCCTACGTGGGCGAGCTTACAACATACACACCACTCCCATCCAAAACCACAATTAAGGTACCAAAATTTGTCTGTCACTGTCACCCAACACTAGCAACCACTGATCTCATTTCTCACACGTTAATTACAAATTACGAACCTTACGTCTAGTTTCATCATCCTCATTCTCCTGATTTTTCTAACCATTAACTTTAAACCACTGATTCTGTTGTCCTTGTTTATTCGTCATTAGATTTTGTTATTTCGATTCATTGTCAAAACTTCAACCTGTAATCGTATAAATATAACTTCACCTGATTAATGCAACCTAGTTTTTGAGTTTTGAGTTGAGCCACACAAGGCACACATTCACACACGCACCAACAACCAATGAAgtcttccacgaagtg

L-1M-U-VuZEP1u-AAA3

cactctgtgaagacTTTACTACTCTATGGGTTTGAATCCTGCTTCAGATTTTCCTACGTGGGCGAGCTTACAACATACACACCACTCCCATCCATGACCACAATTAAGGTACCAAAATTTGTCTGTCACTGTCACCCAACACTAGCAACCACTGATCTCATTTCTCACACGTTAATTACAAATTACGAACCTTACGTCTAGTTTCATCATCCTCATTCTCCTGATTTTTCTAACCATTAACTTTAAACCACTGATTCTGTTGTCCTTGTTTATTCGTCATTAGATTTTGTTATTTCGATTCATTGTCAAAACTTCAACCTGTAATCGTATAAATATAACTTCACCTGATTAAAACAACCTAGTTTTTGAGTTTTGAGTTGAGCCACACAAGGCACACATTCACACACGCACCAACAACCAATGAAgtcttccacgaagtg

Additional Table 1: List of gene fragments and backbones

| **ID** | **Part Name** | **Description** | **Source** | **Reference** |
| --- | --- | --- | --- | --- |
| pICH41388 | GGAG_Pro-CaMV35SShort_TACT | Promoter (0.4 kb), 35s (Cauliflower Mosaic Virus) | Addgene  No. 50253 | Engler et al. 2014 |
| pICH88103 | GGAG_Pro-AtuOcs_5U- AtuOcs _AATG | Promoter + 5'UTR, OCS, (*A. tumefaciens*) | Addgene No. 50273 | Engler et al. 2014 |
| pICH41414 | GCTT_3U+Ter-CaMV35S_CGCT | 3'UTR, polyadenylation signal/terminator, 35s(Cauliflower Mosaic Virus) | Addgene No. 50337 | Engler et al. 2014 |
| pICH41432 | GCTT_3U+Ter -AtuOcs_CGCT | 3'UTR, polyadenylation signal/terminator, OCS (*A. tumefaciens*) | Addgene No. 50343 | Engler et al. 2014 |
| N/A | l-1m-5u-spacer-ccdb | CcdB cassette for dual-fluorescence acceptor | Addgene No. 91061 | Čermák et al., 2017 |
| EC36252 | pL0M-SC-mNeonGreen | mNeonGreen CDS | Synthesis | Shaner et al., 2013 |
| EC36254 | pL0M-SC-tdTomato | tdTomato CDS | Synthesis | Shaner et al., 2004 |
| EC36684 | L-1M-U-AtBRI1 | AtBRI1 5' leader | Synthesis | Si et al. 2020 |
| EC36685 | L-1M-U-AtBRI1-AAA | mutated AtBRI1 5' leader | Synthesis | Si et al. 2020 |
| EC36688 | L-1-U-LsGGP2 | LsGGP2 5’ leader | Synthesis | Si et al. 2020 |
| EC36689 | L-1-U-LsGGP2-AAA | Mutated LsGGP2 5’ leader | Synthesis | Si et al. 2020 |
| EC36686 | L-1M-U-GmPSBSu | GmPSBS 5’ leader, first base C>A to avoid CTG uORF | Synthesis | Glyma.04G249700.1 |
| EC36791 | L-1M-U-GmPSBS-AAA1 | Mutated GmPSBS 5’ leader (ATG to AAA) | Synthesis | Glyma.04G249700.1 |
| EC36790 | L-1M-U-GmPSBS-AAA2 | Mutated GmPSBS 5’ leader (TTG to AAA) | Synthesis | Glyma.04G249700.1 |
| RC00147 | L-1M-GmVDEu | GmVDE 5’ leader | Synthesis | Glyma.19G251000.1 |
| RC00148 | L-1M-GmVDEu-AAA1 | Mutated GmVDE 5’ leader (ATG to AAA) | Synthesis | Glyma.19G251000.1 |
| RC00149 | L-1M-GmVDEu-AAA2 | Mutated GmVDE 5’ leader (ATG to AAA) | Synthesis | Glyma.19G251000.1 |
| RC00150 | L-1M-GmVDEu-AAA3 | Mutated GmVDE 5’ leader (ATG to AAA) | Synthesis | Glyma.19G251000.1 |
| RC00143 | L-1M-GmZEPu | GmZEP 5’ leader | Synthesis | Glyma.17G174500.1 |
| RC00144 | L-1M-GmZEPu-AAA1 | Mutated GmZEP 5’ leader (ATG to AAA) | Synthesis | Glyma.17G174500.1 |
| RC00145 | L-1M-GmZEPu-AAA2 | Mutated GmZEP 5’ leader (ATG to AAA) | Synthesis | Glyma.17G174500.1 |
| RC00146 | L-1M-GmZEPu-AAA3 | Mutated GmZEP 5’ leader (ATG to AAA) | Synthesis | Glyma.17G174500.1 |
| RC00296 | L-1M-U-GmZEPu-CCC1 | Mutated GmZEP 5’ leader (CTG to CCC) | Synthesis | Glyma.17G174500.1 |
| RC00297 | L-1M-U-GmZEPu-CCC2 | Mutated GmZEP 5’ leader (CTG to CCC) | Synthesis | Glyma.17G174500.1 |
| RC00299 | L-1M-U-GmZEPu-TTT1 | Mutated GmZEP 5’ leader (TTG to TTT) | Synthesis | Glyma.17G174500.1 |
| RC00249 | L-1M-U-VuPSBSu | VuPSBS 5’ leader | Synthesis | Vigun09g165900.1 |
| RC00250 | L-1M-U-VuPSBSu-AAA4 | Mutated VuPSBS 5’ leader (ATG to AAA) | Synthesis | Vigun09g165900.1 |
| RC00251 | L-1M-U-VuPSBSu-TTT3 | Mutated VuPSBS 5’ leader (TTG to TTT) | Synthesis | Vigun09g165900.1 |
| RC00252 | L-1M-U-VuPSBSu-TTT4 | Mutated VuPSBS 5’ leader (TTG to TTT) | Synthesis | Vigun09g165900.1 |
| RC00367 | L-1M-U-VuVDE-prim-u | VuVDE 5’ leader | Synthesis | Vigun06g119100.1 |
| RC00400 | L-1M-U-VuVDE-AAA4 | Mutated VuVDE 5’ leader (ACG to AAA) | Synthesis | Vigun06g119100.1 |
| RC00401 | L-1M-U-VuVDE-AAA5 | Mutated VuVDE 5’ leader (ATG to AAA) | Synthesis | Vigun06g119100.1 |
| RC00257 | L-1M-U-VuZEPu | VuZEP 5’ leader | Synthesis | Vigun03g277500.1 |
| RC00258 | L-1M-U-VuZEPu-AAA1 | Mutated VuZEP 5’ leader (ATG to AAA) | Synthesis | Vigun03g277500.1 |
| RC00259 | L-1M-U-VuZEPu-AAA2 | Mutated VuZEP 5’ leader (ATG to AAA) | Synthesis | Vigun03g277500.1 |
| RC00260 | L-1M-U-VuZEPu-AAA3 | Mutated VuZEP 5’ leader (ATG to AAA) | Synthesis | Vigun03g277500.1 |

Additional Table 2: List of vectors

| **ID** | **Figure** reference | **Description** |
| --- | --- | --- |
| RC00214 | Figure 2 | Dual-fluorescence acceptor plasmid (Addgene No. 209019) |
| EC27841 | P19 (fig 1) | Negative control for fluorescence experiments, CaMV35S:P19 expression cassette |
| EC36307 | Dual (fig 1) | Dual-fluorescence positive control |
| EC36795 | Additional fig. 2 | AtuOCS:tdTomato expression cassette |
| EC36796 | Additional fig. 2 | AtuMAS:tdTomato expression cassette |
| EC36797 | Additional fig. 2 | CsVMV:tdTomato expression cassette |
| EC36798 | Additional fig. 2 | StSTLS:tdTomato expression cassette |
| RC00348 | GmPsbS WT (fig 4,6) | Dual-fluorescence reporter; 5’leader: EC36686 |
| RC00349 | GmPsbS *aaa1*(fig 4,6) | Dual-fluorescence reporter; 5’leader: EC36791 |
| RC00350 | GmPsbs *aaa2*(fig 4) | Dual-fluorescence reporter; 5’leader: EC36790 |
| RC00155 | GmVDE WT (fig 4,6) | Dual-fluorescence reporter; 5’leader: RC00147 |
| RC00156 | GmVDE *aaa1* (fig 4,6) | Dual-fluorescence reporter; 5’leader: RC00148 |
| RC00157 | GmVDE *aaa2* (fig 4) | Dual-fluorescence reporter; 5’leader: RC00149 |
| RC00158 | GmVDE *aaa3* (fig 4) | Dual-fluorescence reporter; 5’leader: RC00150 |
| RC00304 | GmZEP WT (fig 4,6) | Dual-fluorescence reporter; 5’leader: RC00143 |
| RC00152 | GmZEP *aaa1* (fig 4,6) | Dual-fluorescence reporter; 5’leader: RC00144 |
| RC00153 | GmZEP *aaa2* (fig 4) | Dual-fluorescence reporter; 5’leader: RC00145 |
| RC00154 | GmZEP *aaa3* (fig 4) | Dual-fluorescence reporter; 5’leader: RC00146 |
| RC00306 | GmZEP *aaa4* (fig 4) | Dual-fluorescence reporter; 5’leader: RC00296 |
| RC00307 | GmZEP *aaa5* (fig 4) | Dual-fluorescence reporter; 5’leader: RC00297 |
| RC00309 | GmZEP *aaa6* (fig 4) | Dual-fluorescence reporter; 5’leader: RC00299 |
| RC00261 | VuPsbs WT (fig 5,6) | Dual-fluorescence reporter; 5’leader: RC00249 |
| RC00262 | VuPsbs *aaa1* (fig 5,6) | Dual-fluorescence reporter; 5’leader: RC00250 |
| RC00263 | VuPsbs *aaa2* (fig 5) | Dual-fluorescence reporter; 5’leader: RC00251 |
| RC00264 | VuPsbs *aaa3* (fig 5,6) | Dual-fluorescence reporter; 5’leader: RC00252 |
| RC00402 | VuVDE WT (fig 5) | Dual-fluorescence reporter; 5’leader: RC00367 |
| RC00404 | VuVDE *aaa1* (fig 5) | Dual-fluorescence reporter; 5’leader: RC00400 |
| RC00405 | VuVDE *aaa2* (fig 5) | Dual-fluorescence reporter; 5’leader: RC00401 |
| RC00269 | VuZEP WT (fig 5) | Dual-fluorescence reporter; 5’leader: RC00257 |
| RC00270 | VuZEP *aaa1* (fig 5) | Dual-fluorescence reporter; 5’leader: RC00258 |
| RC00271 | VuZEP *aaa2* (fig 5) | Dual-fluorescence reporter; 5’leader: RC00259 |
| RC00272 | VuZEP *aaa3* (fig 5) | Dual-fluorescence reporter; 5’leader: RC00260 |

Additional Table 3: RT-qPCR primer sequences and performance parameters. Oligonucleotides used in RT-qPCR experiments, along with their respective binding targets, annealing temperatures (Ta), amplification efficiencies, amplicon size, linear range of detection and corresponding NCBI gene references identified via primer BLAST.

| **ID** | **Sequence** | **Target** | **Orientation** | **Primer-BLAST: *Nicotiana tabacum*** | **T_annealing_ (^o^C)** | **Efficiency (%)** | **Efficiency SE** | **Linear Range (ng RNA)** | **Amplicon size (bp)** |
| --- | --- | --- | --- | --- | --- | --- | --- | --- | --- |
| oliR148 | GACCCTGATGTTGATGTTCGCT | PP2A - Reference | Forward | [XM_016635168.1, XM_016586310.1](https://www.ncbi.nlm.nih.gov/entrez/viewer.fcgi?db=nucleotide&id=1025259103) | 60 | 105.6 | 0.03 | 0.12 - 15.0 | 78 |
| oliR149 | GAGGGATTTGAAGAGAGATTTC | PP2A - Reference | Reverse | [XM_016635168.1, XM_016586310.1](https://www.ncbi.nlm.nih.gov/entrez/viewer.fcgi?db=nucleotide&id=1025259103) | 60 | 105.6 | 0.03 | 0.12 - 15.0 | 78 |
| oliR180 | CGCAGATTGGTGCCGATCTAAA | mNeonGreen - Target | Forward | No additional targets | 60 | 97.9 | 0.012 | 0.04 - 25.0 | 113 |
| oliR181 | TCCTTGCCGTTGATCTGTACCT | mNeonGreen - Target | Reverse | No additional targets | 60 | 97.9 | 0.012 | 0.04 - 25.0 | 113 |

Additional Table 4: List of primers

| **ID** | **Sequence (5' - 3')** | **Application** |
| --- | --- | --- |
| oliR033 | GTGAAAGCCTTCTGCCACTC | Colony PCR & Sanger sequencing of inserted 5' leader; binds upstream of ccdB marker on acceptor RC00214 |
| oliR092 | acgtctacaaagcaagtgg | Colony PCR & Sanger sequencing of inserted 5' leader; binds downstream of ccdB marker on acceptor RC00214 |
| oliR132 | agcatcggtaacatgagc | Sanger sequencing of assembled Loop constructs (LB) |
| oliR022 | GAACCCTGTGGTTGGCATGCACATAC | Sanger sequencing of assembled Loop constructs (RB) |

Additional table 2: R packages

| Package | Version | Citation |
| --- | --- | --- |
| ggplot2 | 3.4.1 | Hadley Wickham, ggplot2: Elegant Graphics for Data Analysis. Springer-Verlag New York, 2016. |
| rstatix | 0.7.2 | Alboukadel Kassambara, rstatix: Pipe-Friendly Framework for Basic Statistical Tests, 2023. |
| dplyr | 1.1.1 | Hadley Wickham, Romain François, Lionel Henry, Kirill Müller and Davis Vaughan, dplyr: A Grammar of Data Manipulation, 2023. |
| reshape2 | 1.4.4 | Hadley Wickham, Reshaping Data with the reshape Package. Journal of Statistical Software, 21(12), 1-20, 2007. |
| ggpubr | 0.6.0 | Alboukadel Kassambara, ggpubr: 'ggplot2' Based Publication Ready Plots, 2023. |
| plotly | 4.10.1 | Carson Sievert, Interactive Web-Based Data Visualization with R, plotly, and shiny. Chapman and Hall/CRC Florida, 2020. |
| RColorBrewer | 1.1.3 | Erich Neuwirth, RColorBrewer: ColorBrewer Palettes, 2022. |
| devtools | 2.4.5 | Hadley Wickham, Jim Hester, Winston Chang and Jennifer Bryan, devtools: Tools to Make Developing R Packages Easier, 2022. |
| readxl | 1.4.3 | Hadley Wickham and Jennifer Bryan, readxl: Read Excel Files, 2023. |
| boot | 1.3-28.1 | Angelo Canty and Brian Ripley, boot: Bootstrap R (S-Plus) Functions, 2023. |

**Additional Note 2: extended methods RT-qPCR**

Per agroinfiltration treatment harboring a construct of interest, ten 6 mm leaf punches were flash-frozen in liquid nitrogen and ground using one 4 mm SPEX stainless steel grinding bead (2150, SPEX SamplePrep, USA) on a TissueLyser Universal Laboratory Mixer-Mill disruptor (85210, QIAGEN, Germany) twice for 90 seconds at 20 Hz with submersion in liquid nitrogen between each grind. RNA was extracted using either NucleoSpin (740933, Macherey Nagel, USA) or RNeasy Plant Mini Kit (74904, QIAGEN, Germany) modified to include four additional RPE buffer wash steps and an on-column RNAse-free DNAse I treatment (79254, QIAGEN, Germany). RNA quantity and quality was checked via NanoDrop One/OneC (ND-ONE-W, ThermoFisher Scientific, USA) and Qubit RNA IQ Assay (Q33221, Life Technologies, USA) on a Qubit 4 Fluorometer (Q33238, Life Technologies, USA). cDNA was synthesized from 8 μl of total RNA and oligo(dT) primers using the Superscript III First Strand Synthesis System (18080051, Invitrogen, USA). cDNA was treated with 2 U E. coli RNAse H (M0523, NEB, USA) for 20 minutes at 37°C. To verify minimal interference from contaminating genomic DNA, no reverse transcriptase (NRT) cDNA samples were prepared by the same protocol with the replacement of reverse transcriptase with nuclease-free water. cDNA was diluted to either 10 or 25 ng/μl equivalent RNA in nuclease-free water.

Primers were designed using the primer wizard on Benchling.com (Benchling, USA) for target gene mNeonGreen and reference gene PP2A (*N. benthamiana*, NCBI: TC21939, Additional table 4). Primer properties including binding T_m_, hairpin T_m_, and ΔG for homo- and heterodimer formation was assessed using Oligo Analyzer (Integrated DNA Technologies, USA) with the following settings: Parameter Sets = qPCR; [Oligo] = 0.33 nm, [$Na^+$] = 50 mM, [Mg^2+^] = 3mM, [dNTP] = 1.2mM. Primers were synthesized and purified by standard desalting (Integrated DNA Technologies, USA). RT-PCR was performed using 500 μM primers, 3 μl diluted cDNA, and SsoAdvanced Universal SYBR Green Supermix (10000076382, Bio-Rad Laboratories, USA) in a 96-well, thin wall hard-shell PCR plate (HSP9601, Bio-Rad Laboratories, USA) sealed with Microseal 'B' (MSB1001, Bio-Rad Laboratories, USA) on a CFX Connect Real-Time PCR Detection System (1855201, Bio-Rad Laboratories, USA) programmed for 95°C for 2 minutes, followed by 39 cycles of 95°C for 10s and 60°C for 30s. Normalized relative quantities (NRQ) were calculated with qBase+ software, version 3.2, using target specific amplification efficiencies (Additional table 4), normalization to a reference gene. The mean Cq value of the three technical replicates was used for statistical analysis using the Kruskal–Wallis test followed by Dunn’s post hoc test, excluding any replicate with a Cq deviation greater than 0.5.

Additional table 5: Colony PCR reaction setup

| **Reagent** | **Volume (ul)** | **Comment** |
| --- | --- | --- |
| Go-Taq Green MM(2x) | 7.5 | M712, Promega, USA |
| Fwd primer (10 uM) | 0.75 |  |
| Rev primer (10 uM) | 0.75 |  |
| H2O | To 15 | No template, colonies dipped directly into Master Mix |
| **Temperature (°C)** | **Time (min)** |  |
| 95 | 2 | Initial denaturation |
| 95 | 0.5 | Denaturation, 30 cycles |
| 60 | 0.5 | Annealing, 30 cycles |
| 72 | 1 min/kb | Extension, 30 cycles |
| 72 | 5 | Final extension |
| 12 | Inf. |  |

Additional table 6: Golden Gate assembly reaction setup

| **Reagent** | **Volume (ul)** | **Comment** |
| --- | --- | --- |
| Total DNA | Up to 5 | Incl. 15 fmol insert + 7.5 fmol acceptor |
| T4 Ligase Buffer (10x) | 2 | M0202L, New England Biolabs, USA |
| IIS enzyme | 1.5 | BpiI (ER1012); BsaI (ER0292); SapI (ER1931); ThermoFisher Scientific, USA |
| T4 ligase | 0.5 | M0202L, New England Biolabs, USA |
| H_2_O | to 20 |  |
| **Temperature (°C)** | **Time (min)** |  |
| 37 | 4 | Cycle up to 20x |
| 16 | 3 |  |
| 50 | 5 |  |
| 65 | 20 |  |
| 12 | Inf. |  |
