# Supplementary material for: A Fluorescence‐Based Transient Expression Assay for the Analysis of Upstream Open Reading Frames in Plants
Source: Plant Direct. 2026 Apr 15;10(4):e70163. doi: 10.1002/pld3.70163 (PMC13084149; doi:10.1002/pld3.70163)
Supplement: Supplementary file 2 — Additional File 2: Testing of tdTomato reference cassettes and power analysis. [file PLD3-10-e70163-s001.docx]

Additional file 2


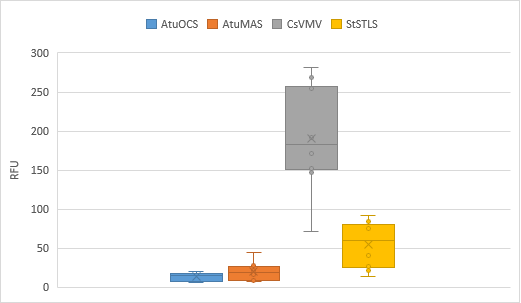


Additional figure 1: Comparison of absolute fluorescence levels from different tdTomato expression cassettes obtained 3 days post agroinfiltration in N. benthamiana (n = 8). AtuOCS, A. tumefaciens octopin synthase promoter; AtuMAS, A. tumefaciens mannopine synthase promoter, CsVMV, cassava vein mosaic virus promoter, StSTLS, S. tuberosum stem- and leaf-specific promoter 1; RFU, Relative Fluorescence Units.

Additional table 1: Mean fluorescence, standard deviation (SD) and coefficient of variance (CV) from different tdTomato expression cassettes obtained 3 days post agroinfiltration in N. benthamiana (n = 8). AtuOCS, A. tumefaciens octopin synthase promoter; AtuMAS, A. tumefaciens mannopine synthase promoter, CsVMV, cassava vein mosaic virus promoter, StSTLS, S. tuberosum stem- and leaf-specific promoter 1; RFU, Relative Fluorescence Units.

| **Promoter** | **Mean (RFU)** | **SD** | **CV** |
| --- | --- | --- | --- |
| AtuOCS | 16.69 | 2.81 | 0.17 |
| AtuMAS | 25.76 | 9.73 | 0.38 |
| CsVMV | 190.83 | 63.77 | 0.33 |
| StSTLS | 55.30 | 28.79 | 0.52 |


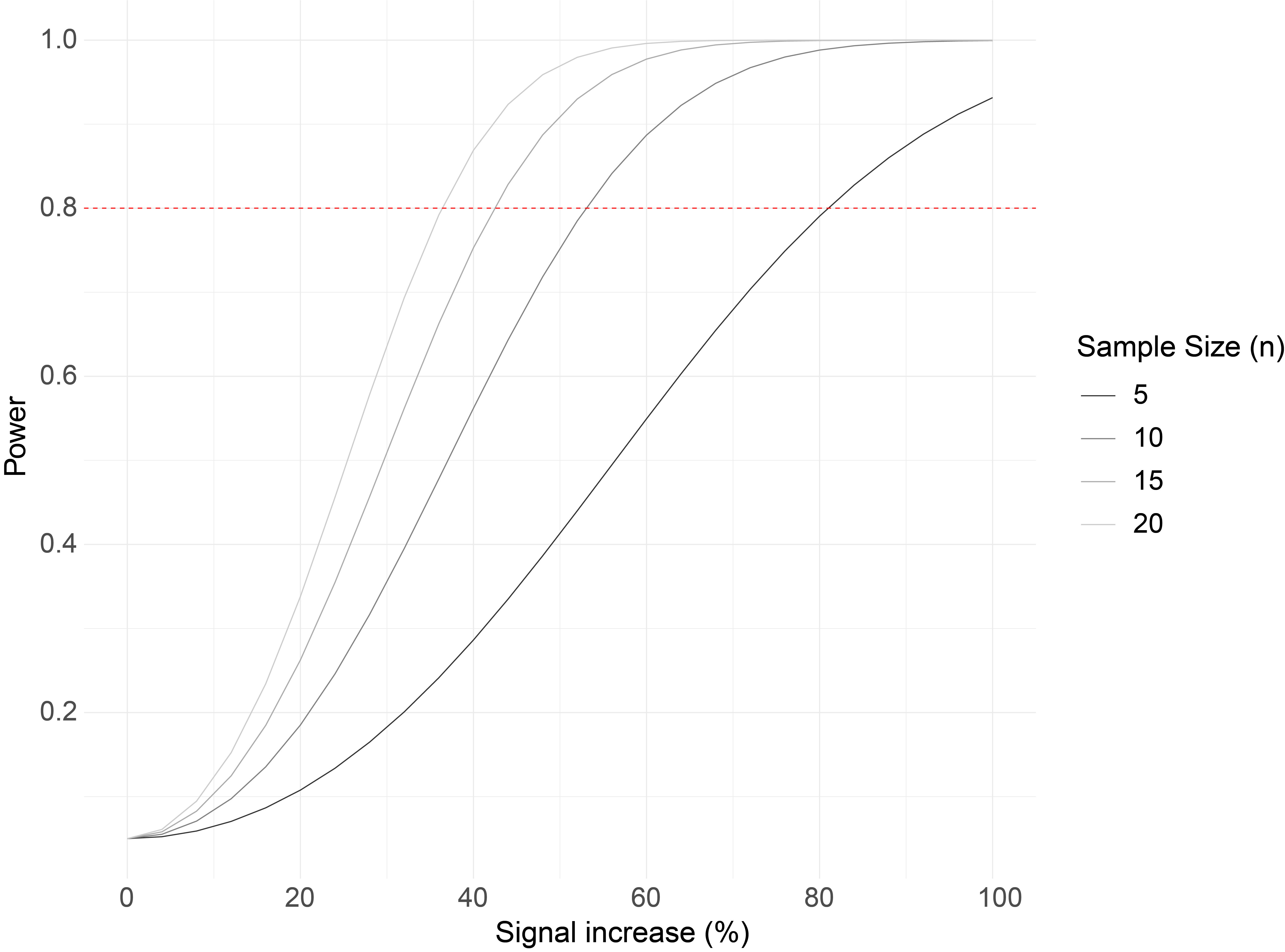


Additional figure 2: Power analysis of a non-parametric bootstrap for two-sample t-testing (α = 0.05) for mean comparison of fluorescence ratios between 5’ leaders. The relationship between the effect size (represented as signal increase) and statistical power across different sample sizes (n) is depicted. The power level of 80% is marked with a red dotted line. The effect sizes were calculated with the standard deviation of dual-fluorescence ratios from the wild-type transcript leader of AtBRI1.


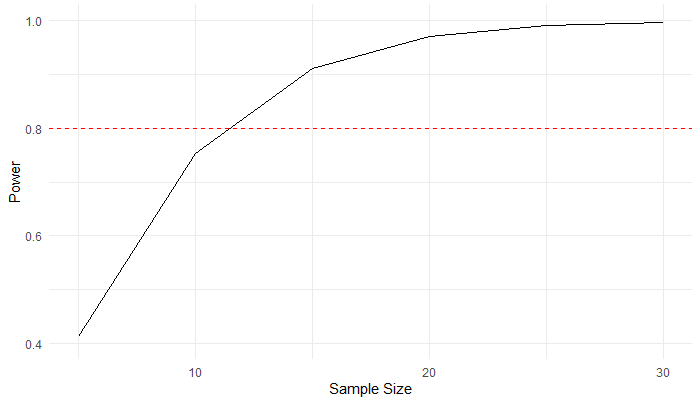


Additional figure 3: Power vs. Sample size of a non-parametric bootstrap for two-sample t-testing (α = 0.05) based on dual-fluorescence ratios from wild-type AtBRI1 and observing a 50% increase in signal.


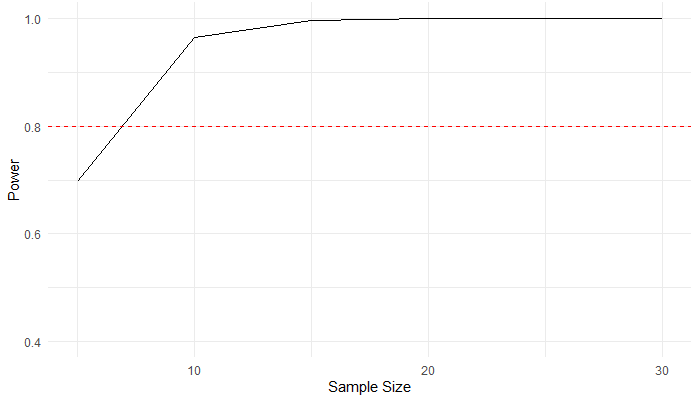


Additional figure 4: Power vs. Sample size of a non-parametric bootstrap for two-sample t-testing (α = 0.05) based on dual-fluorescence ratios from wild-type LsGGP2 and observing a 50% increase in signal.
